# Risk Factors for Lobar and Non-Lobar Intracerebral Hemorrhage in Patients with Vascular Disease

**DOI:** 10.1371/journal.pone.0142338

**Published:** 2015-11-05

**Authors:** Philip H. C. Kremer, Wilmar M. T. Jolink, L. Jaap Kappelle, Ale Algra, Catharina J. M. Klijn, SMART and ESPRIT Study Groups

**Affiliations:** 1 Department of Neurology and Neurosurgery, Brain Center Rudolf Magnus, University Medical Center Utrecht, Utrecht, The Netherlands; 2 Julius Center for Health Sciences and Primary Care, University Medical Center, Utrecht, The Netherlands; 3 Department of Neurology, Donders Institute for Brain, Cognition and Behavior, Centre for Neuroscience, Radboud University Medical Center, Nijmegen, The Netherlands; University of Kansas, UNITED STATES

## Abstract

**Introduction:**

Lobar and non-lobar non-traumatic intracerebral hemorrhage (ICH) are presumably caused by different types of small vessel diseases. The aim of this study was to assess risk factors for ICH according to location.

**Methods:**

In two large prospective studies, SMART (n = 9088) and ESPRIT (n = 2625), including patients with manifest cardiovascular, cerebrovascular or peripheral artery disease or with vascular risk factors, we investigated potential risk factors for ICH during follow-up according to lobar or non-lobar location by Cox proportional hazards analyses.

**Results:**

During 65,156 patient years of follow up 19 patients had lobar ICH (incidence rate 29, 95% CI 19–42 per 100,000 person-years) and 24 non-lobar ICH (incidence rate 37, 95% CI 26–51 per 100,000 person-years). Age significantly increased the risk of lobar ICH (HR per 10 years increase 1.90; 95% CI 1.17–3.10) in the multivariable analysis, but not of non-lobar hemorrhage. Anticoagulant medication (HR 3.49; 95% CI 1.20–10.2) and male sex (HR 3.79; 95% CI 1.13–12.8) increased the risk of non-lobar but not lobar ICH.

**Conclusion:**

This study shows an elevated risk of future ICH in patients with manifestations of, or risk factors for, cardiovascular, cerebrovascular or peripheral artery disease. Our data suggest that risk factors for ICH vary according to location, supporting the hypothesis of a differential pathophysiology of lobar and non-lobar ICH.

## Introduction

Non-traumatic intracerebral hemorrhage (ICH) accounts for 10–20% of all strokes [1]. With a one-month case fatality of 40%, ICH is the most devastating stroke subtype [2]. Of those who survive the initial event, only around 55% recover to function independently after 1 year [3]. In up to 85% of patients, ICH is the result of small vessel disease, which has classically been attributed to hypertensive vasculopathy in non-lobar regions of the brain (basal ganglia, thalamus, cerebellum and brainstem), whereas a large proportion of lobar ICH in elderly patients is attributed to accumulation of amyloid-β in leptomeningeal and cortical blood vessels. Most studies that have investigated risk factors for ICH have focused on ICH in general and did not distinguish between lobar or non-lobar ICH. Age, male sex, hypertension, African-American ancestry and oral anticoagulant medication (OAC) have been found to increase the risk of ICH [4–8]. Diabetes, a known risk factor for small vessel disease, has not emerged as a well-established risk factor for ICH [8], possibly because ICH location was not taken into account [9]. Recently, several case-control studies have suggested variability in risk factors for lobar and non-lobar ICH [9–16], but the variability of risk factors according to ICH location has not been assessed in prospective cohort studies.

We aimed to investigate risk factors for lobar and non-lobar hemorrhage in a large cohort of patients with previous manifestations vascular disease or vascular risk factors.

## Methods

Written informed consent was obtained from all patients or their legal representatives before participating in the study. The institutional ethical committee of the University Medical Center Utrecht (UMCU), The Netherlands, approved the study and informed consent procedure.

### Study population

We included patients from two large prospective studies. In the SMART study (The Second Manifestations of ARTerial disease study) patients with cardiovascular, cerebrovascular and peripheral arterial diseases, renal artery stenosis or abdominal artery aneurysm (AAA), dyslipidemia, diabetes mellitus or hypertension are included and followed for new vascular events [17]. For the current study we included 9088 patients who presented to the UMCU between September 1996 and February 2011. More than 95% of these patients were of Caucasian descent.

The European/Australasian Stroke Prevention in Reversible Ischaemia Trial (ESPRIT) was a randomized clinical trial in which 2739 patients with a TIA or minor ischemic stroke of presumed arterial origin were included between July 1997 and January 2006 in 79 hospitals in 14 countries. ESPRIT compared the combination of acetylsalicylic acid (aspirin) and dipyridamole with aspirin alone for secondary prevention [18]. 2625 patients were eligible for the current analysis after removal of 114 patients who were also included in SMART. Of these patients, 15.2% had Han-Chinese ancestry and 84.8% had Caucasian ancestry.

### Risk factors

In all patients age, sex, blood pressure, hypertension, diabetes, hyperlipidemia, smoking status, and the use of anti-platelet and oral anticoagulant medication (OAC) were recorded at baseline [17, 19]. Additionally, SMART recorded serum glucose levels, body mass index (BMI), mean waist circumference, total-, HDL-, LDL cholesterol levels, triglyceride levels, estimated glomerular filtration rate (eGFR), hyperhomocysteinemia, C reactive protein (CRP), alcohol use and the use of statins [17].

Hypertension was defined as systolic blood pressure ≥160 mmHg or diastolic blood pressure ≥95 mmHg during baseline examination or antihypertensive drug use in SMART [17] and history of hypertension at baseline, based on physician reporting, in ESPRIT [19]. Diabetes mellitus, hyperlipidemia and smoking were defined based on self-reported status in SMART [17] and physician reporting in ESPRIT [19]. Renal function was categorized as severely impaired (eGFR < 50 ml/min); modestly impaired (eGFR 50 ml/min– 80 ml/min); and normal (eGFR > 80 ml/min) [17]. Hyperhomocysteinemia was reported as a dichotomous variable based on the upper limit of the laboratory reference range.

Index events for all patients were categorized as cerebrovascular disease (TIA, minor stroke, stroke, transient monocular blindness and retinal infarctions), cardiac disease, peripheral artery disease (claudication, ischemic renal disease, diabetic vasculopathy, aneurysm of the abdominal artery), risk factors only (diabetes mellitus, hyperlipidemia or hypertension), or other (e.g. asymptomatic carotid artery stenosis).

### Follow-up

Follow-up in SMART consisted of a patient questionnaire regarding hospital admissions and out-patient clinic visits every six months. In ESPRIT, patients were followed by their physician every six months. All outcome events were audited independently by three members of an adjudication committee based on review of medical information and imaging. For all patients in whom a symptomatic intracranial hemorrhage was reported as outcome event two investigators (PHCK, CJMK), who were blinded to the index event and vascular risk factors, reviewed CT and MRI scans to confirm the diagnosis and classify the hemorrhage as lobar or non-lobar. Non-lobar location was defined as hemorrhage in the basal ganglia, internal or external capsule, thalamus, cerebellum or brainstem. All others were considered lobar hemorrhages. In de majority of patients included with TIA/ischemic stroke, CT scan was performed at baseline, and not MRI.

Subarachnoid hemorrhage, subdural- and epidural hematoma and hemorrhage related to cerebral venous sinus thrombosis, vascular malformation or tumor were not included as outcome events. Neither were hemorrhagic transformations of ischemic strokes.

### Data analysis

ICH incidence was calculated by dividing the number of events by the number of person-years during follow-up. A 95% confidence interval (CI) was computed on the basis of a Poisson distribution.

Univariable and multivariable Cox proportional hazards analyses were performed to estimate hazard ratios (HR) and corresponding 95% CIs to assess the association between risk factors at baseline and ICH during follow up. In the multivariable model for ICH overall we adjusted for a maximum of four variables that modified the crude HR by at least 10%. Similarly, risk factors for lobar and non-lobar ICH were adjusted for two variables based on the rule of thumb of one factor per 10 outcome events. Variables present in less than 95% of patients were not included in the multivariable analyses. All analyses were performed with SPSS Statistics (SPSS 20.0; SPSS Inc., Chicago, IL, USA).

## Results

We included 11,713 patients, who were followed up for a total of 65,156 patient years (Table 1 and S1 Table). Median follow-up time was 5.57 years, interquartile range 2.71–7.85 years. Forty-three patients had an ICH during follow up, resulting in an incidence rate of 66 (95% CI 51–84) per 100,000 person-years. In 19 patients (44%) the ICH was lobar (incidence rate 29, 95% CI 19–42 per 100,000 person-years) and in 24 (56%) non-lobar (basal ganglia 16, thalamus 4, cerebellum 2, brainstem 2; incidence rate 37, 95% CI 26–51 per 100,000 person-years).

**Table 1:** Baseline characteristics of patients.

	SMART and ESPRIT	
Number of patients		11713
Age at presentation in years, mean (SD)		58 (12)
Sex, % male		66.8
Caucasian ancestry, %		>95
Systolic blood pressure in mmHg, mean (SD)		144 (22)
Hypertension, %		49.6
Diabetes, %		19.4
Hyperlipidemia, %		50.5
Index event, %	Cerebrovascular event	32.7
	Peripheral artery disease	11.8
	Cardiovascular event	24.8
	Risk factors only	23.9
	Other	6.8
Current smoking, %		32.1
Antiplatelets, %		65.2
Anticoagulant medication, %		6.3
	SMART only	
Number of patients		9088
Serum glucose in mmol/l, mean (SD)		6.3 (2.1)
Body Mass Index in kg/m2, mean (SD)		26.8 (4.4)
Waist circumference in cm, mean (SD)		94 (13)
Index event, %	Cerebrovascular event	13.3
	Peripheral artery disease	15.2
	Cardiovascular event	31.9
	Risk factors only	30.8
	Other	8.8
Total cholesterol in mmol/l, mean (SD)		5.20 (1.40)
Triglycerides in mmol/l, mean (SD)		1.84 (2.02)
HDL cholesterol in mmol/l, mean (SD)		1.25 (0.39)
LDL cholesterol in mmol/l, mean (SD)		3.16 (1.19)
eGFR in ml/min, mean (SD)		93 (33)
Impaired renal function, %	Severely impaired	5.8
	Modestly impaired	31.6
	Normal	61.9
Hyperhomocysteinemia, %		10.9
hsCRP in mg/l, mean (SD)		4.4 (9)
Alcohol use, %	Never	19.7
	Past	9.9
	Recently quit	19.9
	Current	49.8
Statins, %		43.8

For the combined cohort, data were missing in 0.2% of patients or less, except for hyperlipidemia (1.4%). For the SMART cohort data were missing for impaired renal function (0.8%), hyperhomocysteinemia (7.1%), alcohol use (0.7%) and statins in 27.2% of patients. Abbreviations: SMART, Second Manifestations of ARTerial disease study; ESPRIT, European/Australasian Stroke Prevention in Reversible Ischaemia Trial; SD, standard deviation; HDL, high density lipoprotein; LDL, low density lipoprotein; eGFR, estimated glomerular filtration rate; hsCRP, high-sensitivity C-reactive protein.

### All ICH

In the univariable analysis, risk factors for ICH were age (HR per 10 years increase 1.83; 95% CI 1.36–2.45), a cerebrovascular index event (HR 2.98; 95% CI 1.31–6.76), anticoagulant medication (HR 2.95; 95% CI 1.31–6.62), eGFR (HR per 10 ml/min 0.78; 95% CI 0.68–0.89) and renal function (modestly impaired: HR 3.44; 95% CI 1.56–7.58; and severely impaired: HR 8.06; 95% CI 2.93–22.2). We found no association with systolic blood pressure, antiplatelet medication, diabetes or hyperlipidemia (S2 Table). In the multivariable model, mean age (HR per 10 year increase 1.58; 95% CI 1.16–2.15) and anticoagulant medication (HR 2.75; 95% CI 1.02–7.42) remained significantly associated with ICH (Table 2).

**Table 2:** Multivariable hazard ratio’s for risk factors for intracerebral hemorrhage in general, lobar and non-lobar hemorrhage.

SMART and ESPRIT	All ICH		Lobar ICH		Non-lobar ICH	
	aHR (95% CI)	Adjusted for	aHR (95% CI)	Adjusted for	aHR (95% CI)	Adjusted for
Age (per 10 years increase)	1.58 (1.16–2.15)	I	1.90 (1.17–3.10)	I	1.31 (0.86–1.99)	I, S
Male sex	1.54 (0.74–3.17)	I	0.77 (0.29–2.01)	P, I	3.79 (1.13–12.8)	S
Systolic blood pressure (per 10 mmHg)	0.97 (0.84–1.12)	A, I	0.83 (0.66–1.05)	A, I	1.08 (0.90–1.30)	A, I
Hypertension	0.97 (0.51–1.85)	A, I	0.54 (0.21–1.41)	A, I	1.59 (0.64–4.00)	A, I
Index event						
Cerebrovascular	2.28 (0.78–6.67)	A, P	1.12 (0.24–5.30)	A, P	4.17 (0.94–18.5)	A, P
Peripheral artery disease	1.05 (0.35–3.18)	A, P	0.84 (0.18–4.00)	A, P	1.29 (0.27–6.12)	A, P
Cardiovascular	0.51 (0.13–1.93)	A, P	0.55 (0.10–3.04)	A, P	0.34 (0.03–3.54)	A, P
Risk factors only	Reference		Reference		Reference	
Antiplatelets	0.96 (0.38–2.39)	A, I, C, G	0.99 (0.28–3.55)	A, I	0.92 (0.25–3.35)	I, C
Anticoagulant medication	2.75 (1.02–7.42)	A, I, P	1.46 (0.31–6.79)	A, P	3.49 (1.20–10.2)	A, P
SMART only	All ICH		Lobar ICH		Non-lobar ICH	
	aHR (95% CI)	Adjusted for	aHR (95% CI)	Adjusted for	aHR (95% CI)	Adjusted for
eGFR (per 10 ml/min)	0.92 (0.77–1.10)	A, I	0.92 (0.72–1.17)	A	0.94 (0.74–1.21)	A, S
Impaired renal function						
Severe	2.78 (0.85–9.09)	A, S, I	2.45 (0.48–12.5)	A, I	3.22 (0.58–17.9)	A, S
Moderate	1.26 (0.49–3.25)	A, S, I	1.01 (0.27–3.70)	A, I	2.09 (0.54–8.04)	A, S
Normal	Reference		Reference		Reference	
Hyperhomocysteinemia	1.63 (0.63–4.17)	A, S, I	0.93 (0.20–4.29)	A, I	2.64 (0.75–9.25)	A, I

The number of patients included in the analyses varied from 8,453 to 11,643 in the combined cohort and from 7,232 to 8,953 in the SMART cohort. Abbreviations: SMART, Second Manifestations of ARTerial disease study; ESPRIT, European/Australasian Stroke Prevention in Reversible Ischaemia Trial; ICH, intracerebral hemorrhage; aHR, adjusted Hazard Ratio; SBP, systolic blood pressure; eGFR, estimated glomerular filtration rate; I, index event; A, age; P, antiplatelet medication; C, anticoagulant medication; G, sex; S, systolic blood pressure.

### Lobar ICH

In the univariable analysis we found age (HR per 10 years increase 2.09; 95% CI 1.31–3.32), eGFR (HR per 10 ml/min 0.79; 95% CI 0.65–0.95) and renal function (severely impaired: HR 6.69; 95% CI 1.67–26.8) to be associated with lobar ICH (S1 Table). In the multivariable model, only age remained associated (HR per 10 years increase 1.90; 95% CI 1.17–3.10) (Table 2).

### Non-lobar ICH

For non-lobar ICH, we found age (HR per 10 years increase 1.66; 95% CI 1.13–2.43), male sex (HR 3.50; 95% CI 1.04–11.7), systolic blood pressure (HR per 10 mmHg 1.20; 95% CI 1.02–1.40), hypertension (HR 2.51; 95% CI 1.04–6.06), a cerebrovascular index event (HR 3.63; 95% CI 1.18–11.2), anticoagulant medication (HR 4.00; 95% CI 1.50–10.7), eGFR (HR per 10 ml/min 0.77; 95% CI 0.63–0.93) and renal function (modestly impaired: HR 4.87; 95% CI 1.50–15.8; severely impaired: HR 10.1; 95% CI 2.26–45.2), and hyperhomocysteinemia (HR 3.68; 95% CI 1.11–12.2) as risk factor in the univariable analysis (S1 Table). In the multivariable models male sex (HR 3.79; 95% CI 1.13–12.8) and anticoagulant medication (HR 3.49; 95% CI 1.20–10.2) remained strong and independent risk factors (Table 2).

## Discussion

This prospective cohort study shows an elevated risk of ICH in patients with manifestations of arterial disease or vascular risk factors with an incidence rate of 66 per 100,000 person-years. Our study shows that increasing age is the most important independent risk factor for lobar ICH, whereas the risk of lobar ICH is similar in men and women. For non-lobar ICH male sex, but not increasing age is an independent risk factor. Oral anticoagulant medication was an independent risk factor for non-lobar ICH, but not for lobar ICH.

The incidence rate of ICH in our cohort is almost twice that of the comparable age-group in a recent meta-analysis on the incidence of ICH in population based studies (incidence rate 36.5; 95% CI 28.4–46.7 per 100,000 person-years in the age group 55–64 years) [2]. This finding supports the notion that manifestations of arterial ischemic diseases in any vascular bed throughout the body and ICH share etiological factors. In the SMART cohort, the incidence of ischemic stroke was 427 per 100,000 person-years (95% CI 387–469) and the incidence of myocardial infarction was 677 per 100,000 person-years (95% CI 627–730) [20]. In contrast to previous case-control studies, we were also able to investigate age and sex as independent risk factors. Our study suggests that the effect of increasing age on the risk of ICH is larger for lobar than for non-lobar ICH. This finding is consistent with the increasing proportional incidence of lobar ICH in increasing age-bands in a recent population-based study in the Dijon area in France [13]. The increasing proportion of lobar ICH with increasing age is probably associated with a relative high incidence of cerebral amyloid angiopathy in elderly patients [15].

Our finding that male sex is a risk factor for non-lobar ICH and not lobar ICH is in line with the results of a previous population based study [14]. Hypertension is a well-recognized risk factor for ICH in general [6, 8], with a stronger association with non-lobar than with lobar ICH [9, 21]. A recent, large population based study found hypertension to be associated with increased risk of non-lobar ICH (attributable risk 48.4%) but not with lobar ICH [11]. A possible explanation for our finding that hypertension was not independently associated with lobar or non-lobar ICH is that in our cohort patients were included because of manifest vascular disease or multiple vascular risk factors; in our cohort 50% of patients had hypertension. In a recent analysis of three population-based cohort studies hypertension was strongly associated with ICH overall but not specified for ICH location [22]. In these cohorts the proportion of patients on antihypertensive medication was lower than in our study (between 25 and 46%) [22]. Furthermore, variation in the applied definition of hypertension may contribute to the differences in the observed associations [21]. Finally, there may have been index-event bias [23].

One recent study found that anticoagulant medication was associated with increased risk of lobar but not non-lobar hemorrhage [10]. An older study reported similar findings [24]. Other studies showed that use of anticoagulant medication is not independently associated with hemorrhage location [25,26], or specifically increased the risk of cerebellar hemorrhage [27]. In contrast, we found that anticoagulant medication significantly increased the risk of non-lobar ICH but not lobar ICH. Multiple factors may contribute to the disparate results. First, and probably most important, the absolute number of ICH patients on anticoagulant medication in the other studies as well as ours was generally small. Second, definitions of lobar, non-lobar and deep hemorrhage vary between studies. Finally, patients in our cohort were younger than in most other reports.

Impaired renal function as well as lower eGFR was associated with ICH in univariable analyses, but these associations were no longer present after adjustment for age. Poor kidney function is increasingly recognized as an independent risk factor for stroke [28], but has been studied specifically in only few studies of ICH [28,29]. We could not corroborate these findings in our multivariable analysis.

While diabetes did not emerge as a risk factor for ICH in general [6,8], it has been independently associated with non-lobar ICH in a recent case-control study [9]. Our results do not corroborate with this finding. Possibly, because in our cohort of patients with arterial manifestations of arterial disease or vascular risk factors, hyperglycemia has been regulated relatively rigorously.

A recent case-control study showed history of stroke to be associated with risk of non-lobar and lobar ICH [11]. In this study, cases where age-matched to controls. We could not confirm this finding in the multivariable analysis including age.

Hyperhomocysteinemia was found to be associated with ICH in a retrospective observational study from China [30]. In that study, lobar versus non-lobar hemorrhage location was not reported. The association remained statistically significant after adjusting for hypertension and other possible confounders. In our study, hyperhomocysteinemia was associated with non-lobar hemorrhage in the univariable analysis only. Adjustment for age and index event negated the association. The association with hypertension followed a similar pattern.

Our study has strengths as well as limitations. Strengths are that we could combine data from two large prospective cohorts of high-risk patients and that we determined ICH location blinded to risk factors. Our study is the first prospective cohort study to investigate risk factors according to location, allowing us to also assess the effect of age and sex. Limitations are that some risk factors were not available in the ESPRIT patients, that insufficient data was available to control for antihypertensive drug use, that we had no information on level of education [5,11], and that we had no information on APOE genotype. Furthermore, microbleeds and leukoaraiosis at baseline could not be assessed because of limited availability of MRI-scans and we did not have pathological information to determine the probability of CAA in patients with lobar ICH. Finally, despite the large cohort that we studied and the relatively high incidence of ICH in this cohort, the total number of patients with ICH was still limited.

In conclusion, our study lends further support to the concept that the underlying vascular disease in patients with lobar and non-lobar spontaneous ICH differ. Patients with manifestations of arterial disease or vascular risk factors are at relatively high risk of ICH. Future studies of etiological factors for ICH should distinguish effects for lobar and non-lobar ICH. Further understanding of the characteristics of the underlying vascular disease may help to determine more specific treatment options for patients with lobar and non-lobar ICH.

## Supporting Information

S1 Table Univariable hazard ratio’s for risk factors for intracerebral hemorrhage in general, in lobar and non-lobar hemorrhage. The number of patients included in the analyses for each of the variables varied from 8,453 to 11,643 in the combined cohort and from 7,232 to 8,953 in the SMART cohort. Abbreviations: SMART, Second Manifestations of ARTerial disease study; ESPRIT, European/Australasian Stroke Prevention in Reversible Ischaemia Trial; ICH, intracerebral hemorrhage; HDL, high density lipoprotein; LDL, low density lipoprotein; eGFR, estimated glomerular filtration rate; hsCRP, high-sensitivity C-reactive protein.
(DOCX)

S2 Table Baseline characteristics of patients, SMART en ESPRIT separately. (DOCX)
