# Supplementary material for: Risk Factors for Lobar and Non-Lobar Intracerebral Hemorrhage in Patients with Vascular Disease
Source: PLoS One. 2015 Nov 5;10(11):e0142338. doi: 10.1371/journal.pone.0142338 (PMC4634984; doi:10.1371/journal.pone.0142338)
Supplement: S1 Table — The number of patients included in the analyses for each of the variables varied from 8,453 to 11,643 in the combined cohort and from 7,232 to 8,953 in the SMART cohort. Abbreviations: SMART, Second Manifestations of ARTerial disease study; ESPRIT, European/Australasian Stroke Prevention in Reversible Ischaemia Trial; ICH, intracerebral hemorrhage; HDL, high density lipoprotein; LDL, low density lipoprotein; eGFR, estimated glomerular filtration rate; hsCRP, high-sensitivity C-reactive protein. (DOCX) [file pone.0142338.s001.docx]

**Supplemental Table 1. Baseline characteristics of patients, SMART en ESPRIT separately**

|  |  | | SMART | | ESPRIT |
| --- | --- | --- | --- | --- | --- |
| Number of patients | | | | 9,088 | 2,625 |
| Age at presentation in years, mean (SD) | | | | 56 (12) | 63 (11) |
| Sex, % male | | | | 67.4 | 65.0 |
| Systolic blood pressure in mmHg, mean (SD) | | | | 142 (21) | 152 (24) |
| Hypertension, % | |  | | 46.9 | 59.2 |
| Diabetes, % | |  | | 19.7 | 18.7 |
| Hyperlipidemia, % | |  | | 51.9 | 45.6 |
| Index event, % | | Cerebrovascular event | | 13.3 | 100.00 |
|  |  | Peripheral artery disease | | 15.2 | 0 |
|  |  | Cardiovascular event | | 31.9 | 0 |
|  |  | Risk factors only | | 30.8 | 0 |
|  |  | Other | | 8.8 | 0 |
| Current Smoking, % | |  | | 30.8 | 36.3 |
| Antiplatelets, % | |  | | 55.1 | 100 |
| Anticoagulant medication, % | |  | | 8.1 | 0 |
