# Supplementary material for: Risk Factors for Lobar and Non-Lobar Intracerebral Hemorrhage in Patients with Vascular Disease
Source: PLoS One. 2015 Nov 5;10(11):e0142338. doi: 10.1371/journal.pone.0142338 (PMC4634984; doi:10.1371/journal.pone.0142338)
Supplement: S2 Table — (DOCX) [file pone.0142338.s002.docx]

|  | SMART and ESPRIT  *All ICH* (n=43) | SMART and ESPRIT  *Lobar* (n=19) | SMART and ESPRIT  *Non-lobar* (n=24) |
| --- | --- | --- | --- |
| Age (per 10 years increase) | 1.83 (1.36-2.45) | 2.09 (1.31-3.32) | 1.66 (1.13-2.43) |
| Male sex | 1.66 (0.82-3.37) | 0.87 (0.34-2.21) | 3.50 (1.04-11.7) |
| Systolic blood pressure (per 10 mmHg) | 1.12 (0.99-1.27) | 1.01 (0.82-1.24) | 1.20 (1.02-1.40) |
| Hypertension | 1.61 (0.87-2.96) | 0.96 (0.39-2.38) | 2.51 (1.04-6.06) |
| Diabetes | 0.79 (0.35-1.76) | 1.08 (0.36-3.24) | 0.58 (0.17-1.93) |
| Hyperlipidemia | 0.81 (0.44-1.50) | 1.14 (0.44-2.94) | 0.63 (0.28-1.42) |
| Index event  Cerebrovascular event  Peripheral artery disease  Cardiovascular event  Risk factors only | 2.98 (1.31-6.76)  1.53 (0.53-4.42)  0.58 (0.18-1.94)  Reference | 2.28 (0.67-7.70)  1.52 (0.34-6.80)  0.89 (0.20-3.98)  Reference | 3.63 (1.18-11.2)  1.54 (0.35-6.89)  0.29 (0.03-2.58)  Reference |
| Current smoking | 0.88 (0.46-1.69) | 0.93 (0.35-2.45) | 0.84 (0.35-2.03) |
| Antiplatelets | 1.34 (0.71-2.53) | 1.65 (0.62-4.37) | 1.15 (0.50-2.65) |
| Anticoagulants | 2.95 (1.31-6.62) | 1.77 (0.41-7.68) | 4.00 (1.50-10.7) |
|  | SMART  *All ICH* (n=32) | SMART  *Lobar* (n=16) | SMART  *Non-lobar* (n=16) |
| Serum glucose level (mmol/l) | 1.03 (0.89-1.18) | 1.04 (0.86-1.25) | 1.01 (0.83-1.25) |
| Body mass index (kg/m^2^) | 1.01 (0.93-1.09) | 1.00 (0.90-1.12) | 1.01 (0.91-1.13) |
| Waist circumference (per 10 cm) | 1.22 (0.89-1.69) | 1.27 (0.84-1.33) | 1.16 (0.70-1.93) |
| Total cholesterol (mmol/l) | 1.04 (0.82-1.32) | 1.16 (0.88-1.52) | 0.90 (0.61-1.32) |
| Triglycerides (mmol/l) | 0.73 (0.49-1.09) | 0.84 (0.53-1.35) | 0.59 (0.29-1.17) |
| HDL cholesterol (mmol/l) | 1.20 (0.51-2.81) | 0.99 (0.28-3.55) | 1.41 (0.45-4.44) |
| LDL cholesterol (mmol/l) | 1.14 (0.86-1.51) | 1.34 (0.94-1.91) | 0.94 (0.60-1.46) |
| Hyperhomocysteinemia | 2.20 (0.88-5.49) | 1.22 (0.27-5.46) | 3.68 (1.11-12.2) |
| eGFR (per 10 ml/min) | 0.78 (0.68-0.89) | 0.79 (0.65-0.95) | 0.77 (0.63-0.93) |
| Impaired renal function  Severely impaired  Modestly impaired    Normal | 8.06 (2.93-22.2)  3.44 (1.56-7.58)  Reference | 6.69 (1.67-26.8)  2.49 (0.84-7.40)  Reference | 10.1 (2.26-45.2)  4.87 (1.50-15.8)  Reference |
| Alcohol status    Current  Recently quit  Past  Never | 0.72 (0.26-2.03)  0.82 (0.31-2.18)  2.24 (0.79-6.40)  Reference | 0.41 (0.09-1.78)  0.56 (0.16-1.97)  1.36 (0.32-5.67)  Reference | 1.45 (0.28-7.58)  1.50 (0.29-7.81)  4.44 (0.81-24.3)  Reference |
| hsCRP (per 10 mg/L) | 1.13 (0.89-1.44) | 0.98 (0.51-1.88) | 1.20 (0.95-1.52) |
| Statins | 1.57 (0.47-5.23) | 1.08 (0.24-4.87) | 2.98 (0.33-26.7) |

**Supplemental Table 2. Univariable hazard ratio’s for risk factors for intracerebral hemorrhage in general, in lobar and non-lobar hemorrhage.**

The number of patients included in the analyses for each of the variables varied from 8,453 to 11,643 in the combined cohort and from 7,232 to 8,953 in the SMART cohort. Abbreviations: SMART, Second Manifestations of ARTerial disease study; ESPRIT, European/Australasian Stroke Prevention in Reversible Ischaemia Trial; ICH, intracerebral hemorrhage; HDL, high density lipoprotein; LDL, low density lipoprotein; eGFR, estimated glomerular filtration rate; hsCRP, high-sensitivity C-reactive protein.
